# Food resource partitioning between juvenile and mature weatherfish *Misgurnus fossilis*

**DOI:** 10.1002/ece3.7340

**Published:** 2021-03-13

**Authors:** Kacper Pyrzanowski, Grzegorz Zięba, Joanna Leszczyńska, Małgorzata Adamczuk, Małgorzata Dukowska, Mirosław Przybylski

**Affiliations:** ^1^ Department of Ecology and Vertebrate Zoology Faculty of Biology and Environmental Protection University of Lodz Lodz Poland; ^2^ Department of Hydrobiology and Protection of Ecosystems Faculty of Environmental Biology University of Life Sciences in Lublin Lublin Poland

**Keywords:** diet preference, habitat use, ontogenetic niche shift

## Abstract

This study represents a description of the diet composition of one of the largest European cobitids, the weatherfish *Misgurnus fossilis*. Specimens were collected in a drainage canal, representing a typical habitat for weatherfish, and with gut content analysis conducted with regard to individual total length and maturity stage. Overall, the weatherfish diet mainly consisted of Copepoda, Cladocera, Ostracoda, Oligochaeta, *Asellus aquaticu*s, Chironomidae and Coleoptera larvae, Gastropoda, and detritus. To evaluate size‐related patterns of resource use, fish were assigned to two size classes, defined according to size at first maturation. ANOSIM analyses revealed major ontogenetic shifts in feeding strategy, which were related to size and maturity, with a significant ontogenetic shift in feeding pattern, marked by differences in the proportions of the main taxonomic groups of prey consumed. Copepoda and Cladocera dominated in the diet of small and immature individuals, while large weatherfish primarily fed on detritus. Similarly, cluster analysis of diet classified into these food types showed distinct two groups comprising juvenile and mature fish. The weatherfish is a food opportunist using all available resources, but spatially showed a change in feeding sites. Smaller and sexually immature individuals more often use prey caught in the water column and among macrophytes, while larger (sexually mature) individuals occupying the bottom, much more often use detritus as a food base.

## Graphical abstract

Despite the clear habitat specialization, the weatherfish is an opportunistic species, using all available food resources. Significant food niche overlapping between fish size groups may not indicate the putative competition between juveniles and matured fish. However under optimal environmental conditions, juvenile fish experience an ontogenetic shift from water column prey to ultimate and preferred by mature weatherfish bottom substrate prey.

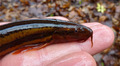

## 1 INTRODUCTION

**FIGURE 1:**
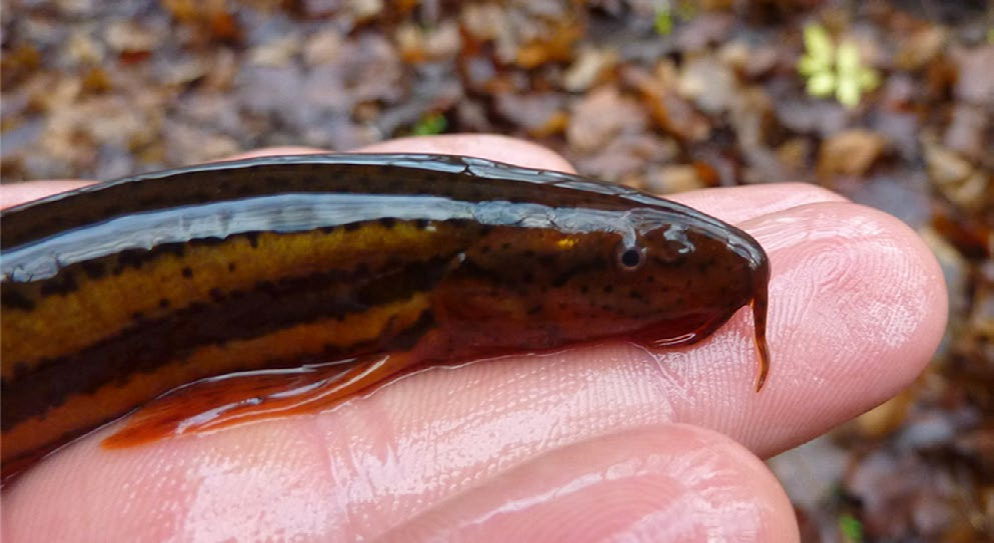
Weatherfish (*Misgurnus fossilis*). Photograph taken by Grzegorz Zięba

The weatherfish *Misgurnus fossilis* (Fig. 1) is a benthic cobitid widely distributed in Eurasian lowland reaches of slow‐flowing rivers, canals, drainage ditches, oxbows, unmanaged lakes, and ponds (Meyer and Hinrichs 2000; Kottelat and Freyhof 2007; Pekarik et al. 2008). This species tolerates a relatively wide spectra of environmental conditions, but typical habitats are waterbodies with a thick organic substrate and dense macrophytes. Weatherfish can tolerate unfavorable environmental conditions, such as low dissolved oxygen concentrations (Jakubowski 1958; Drozd et al. 2009), high water temperatures, and a scarcity of prey (Pyrzanowski et al. 2019). Weatherfish can survive in waterbodies with relatively high levels of pollution (Pyrzanowski et al. 2021 *in press* **)** and unstable habitats exposed to short‐term droughts (Pyrzanowski et al. 2020a), a consequence of their ability to burrow into soft mud during dry periods (Boroń et al. 2002). In recent decades, populations of weatherfish have declined in response to habitat deterioration (Belle et al. 2017). Although locally abundant, across Europe weatherfish are rare and threatened, though currently classified as species of low concern (LC) (Freyhof and Brook 2011). *M. fossilis* is listed in the European Fauna‐Flora‐Habitat and Natura 2000 directives (Annex II of the Council Directive 92/43/EEC), representing species of European Community interest (E.U. 1992).

As a benthic species, with small eyes and mouth, *M. fossilis* feed primarily on aquatic insects, particularly the larvae of Chironomidae, Coleoptera, Ephemeroptera, as well as on Crustacea, Mollusca, and zooplankton (Boroń et al. 2002; Pyrzanowski et al. 2019). In unfavorable environmental conditions, with restricted food resources, detritus can contribute a major component of the diet (Pyrzanowski et al. 2019). A primary requirement for the effective protection of threatened species is an understanding of their life history (Kirchhofer et al. 1996). In the case of weatherfish, these data are still largely lacking. There are few published studies on the biology of *M. fossilis,* with limited focus on threats and protection (Hartvich et al. 2010; Freyhof and Brooks 2011; Schreiber et al. 2018a), habitat preferences (Meyer and Hinrichs 2000; Pyrzanowski et al. 2015), reproduction (Geldhauser 1992; Adamkova‐Stibranyiova et al. 1999; Drozd et al. 2009; Schreiber et al. 2017, 2017a; Pyrzanowski et al. 2021 *in press*), growth (Pyrzanowski et al. 2020b), and morphology (Kotusz 1996). Recently, several studies have been published suggesting the usefulness of weatherfish as a new species for studies of the toxicity of aquatic ecosystems (Schreiber et al. 2017b, 2018b). Despite some general reports on the food and feeding habits of weatherfish, detailed information is scarce with results restricted to descriptions of diet composition but typically without an analysis of feeding strategy or feeding niche. The aim of the current study was to investigate the weatherfish foraging strategy under favorable conditions; when food resources were abundant, with no oxygen deficit, and at a temperature when fish were able to accumulate reserves for growth and reproduction. An additional aim was to identify whether food resources might be partitioning between juvenile and mature individuals.

## 2 MATERIALS AND METHODS

**FIGURE 2:**
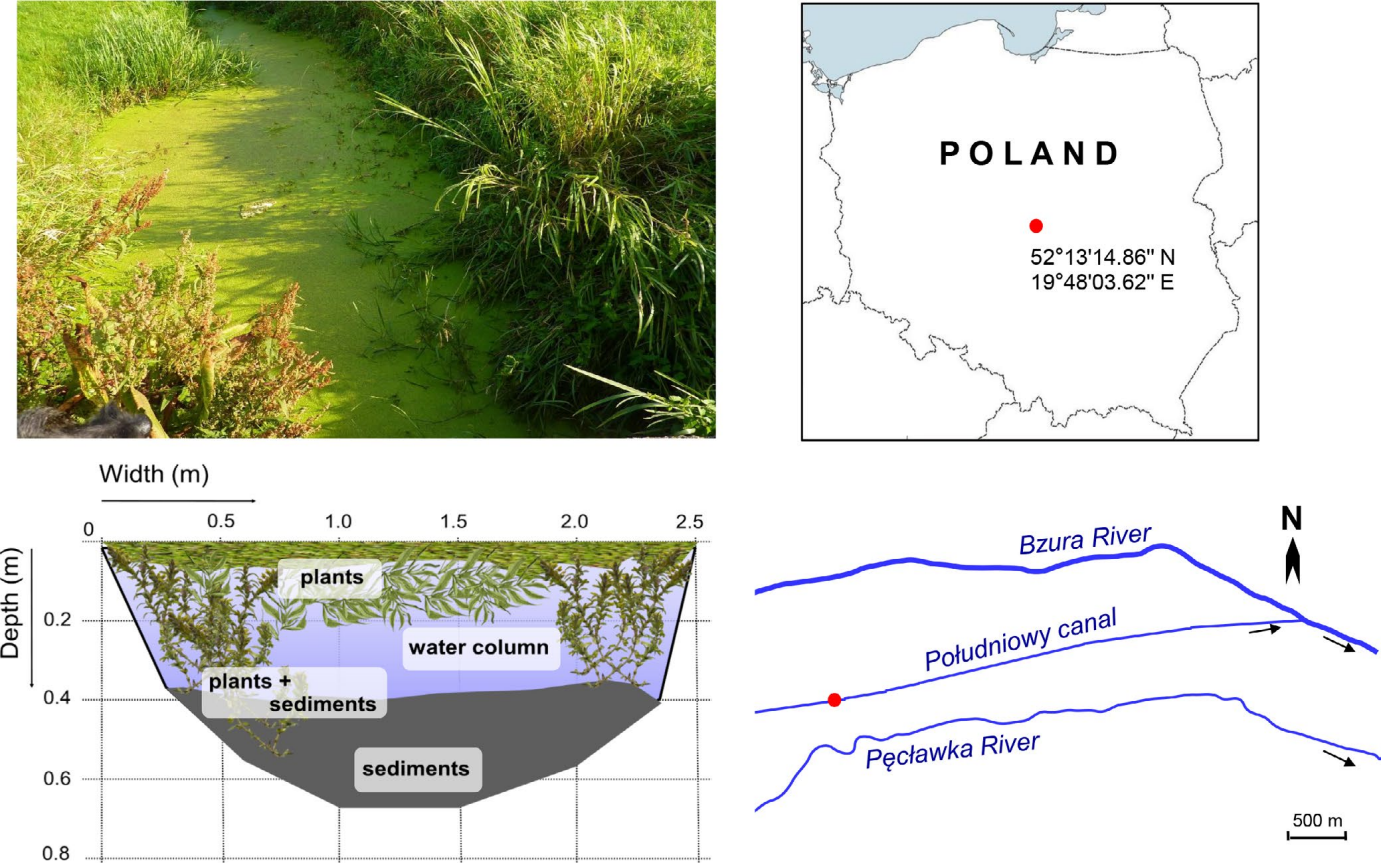
Study area

The study was carried out in the Południowy canal (52°13'14.86'' N; 19°48'03.62'' E), a tributary of the River Bzura with a total length of 6.5 km and an average slope of 0.41‰. The canal is a part of a drainage network of the Natura 2000 Bzura‐Ner glacial valley (PLH100006). The catchment is typically agricultural and dominated by grazing meadows. The average width of the canal is about 2.5–3.0 m, and the average depth varies from 0.3 m to 0.8 m. The substrate consists of sand covered with organic sediments and is overgrown with submerged and emergent vegetation (Fig. 2). The Południowy canal is an example of a site in which the occurrence and abundance of weatherfish has been recognized as high (Pyrzanowski et al. 2015). The fish assemblage of the investigated stretch of the Południowy canal comprised a total of only 5 species, with weatherfish the dominant species and a low abundance of undersized specimens of pike (*Esox lucius*), crucian carp (*Carassius carassius*), roach (*Rutilus rutilus*), and tench (*Tinca tinca*).

A total of 64 weatherfish, ranging from 8.7 to 20.5 cm in total length (TL), were collected in May 2015 using pulsed backpack electrofishing equipment (EFGI 650, BSE Bretschneider Spezialelektronik, Chemnitz, Germany). Captured fish were killed using clove oil and preserved in 10% formaldehyde (Javahery 2012). As weatherfish are a protected species in Poland, fish collection was conducted under permission from the Local Ethics Committee (66/ŁB729/2014) and the Regional Directorate of Environmental Protection (WPN‐II.6401.268.2014.KW2).

Each specimen was measured for total length (TL) to the nearest 0.1 cm and weighed (W) to the nearest 0.01 g. Each fish was dissected to remove the alimentary tract and permit visual examination of the gonads for sex determination. Gut contents were weighed to the nearest 1 mg and stored in glycerine. Among the fish examined, only 5 specimens were found with an empty gut and were consequently excluded from the dataset. Food items were subsequently identified to the lowest practical taxon; that is, to order, family or species and/or genus where possible, under a stereomicroscope (Nikon SMZ1000) and counted (volumetric method) (Hyslop 1980). The total number and estimated weight of each food item were recorded for each fish.

To assess ontogenetic changes in fish diet composition, weatherfish were separated into juvenile and mature fish on the basis of visual gonad examination. These two groups represent size classes; that is, small—juveniles (TL≤12.0 cm) and large—mature (TL>12.0 cm), corresponding with size at first maturation. It was assumed that the smaller juvenile class corresponded with ages of 2+ and 3+, and the larger mature class of specimens at an age from 3+ to 5+ (Pyrzanowski et al. 2020b). Since sampling was conducted in May during the reproductive season, young‐of‐the‐year individuals were not included in the sample.

Prey items were combined by taxon and quantified by the frequency of occurrence (%FO*_i_*) and percentage of biomass (%W*_i_*) (Hyslop 1980). Estimates were made of the gut fullness coefficient (FC), defined as the proportion (in %) of gut content weight to fish weight. The importance of each category within the diet was also estimated using the Index of Relative Importance (IRI) (Pinkas et al. 1971) and its standardized value (%IRI; Cortés 1997), estimated as follows:

$${\text{IRI}}_{i}=\%F0_{i}\left(\%W_{i}+\%N_{i}\right)$$

or

$$\text{IRI}=\%{\text{FO}}_{i}\times \%W_{i}\;\text{as}\;f\;\%\;N_{i}\;\text{is}\;\text{not}\;\text{estimated}\;\text{for}\;\text{food}\;\text{items}\;\text{in}\;\text{our}\;\text{study}$$

and

$$\%{\text{IRI}}_{i}=100\;{\text{IRI}}_{i}/\sum {\text{IRI}}_{i}$$

To compare the overall diet composition according to fish size classes, a one‐way permutation analysis of similarity (ANOSIM, Bray‐Curtis similarity coefficient) was used. ANOSIM is analogous to an ANOVA procedure, with a non‐parametric permutation applied to a rank similarity matrix of samples. In this procedure, the R statistic provides an absolute measure of how groups are separated (Clarke 1993). Generally, R values range between 0 and 1+, indicating no and complete separation between groups, respectively (Clarke and Warwick 1994). The significance level of the R statistics was calculated using a procedure including 9999 permutations of the dataset.

The similarity percentage procedure (SIMPER) was used to identify which food categories were most likely responsible for the patterns detected by ANOSIM. SIMPER provided the average dissimilarities between the fish size classes and identified which prey categories made the greatest contribution to any dissimilarities between size classes (Clarke and Warwick 1994).

Dietary niche of weatherfish size class was characterized as trophic diversity indices: food category richness (S), Simpson’s index of dominance (D), Levin’s (B), Shannon‐Wiener’s (Hʼ), and their standardized forms (evenness indices), Ba and Jʼ, as food niche width. All these indices were defined as follows:

S‐thenumberoffoodcategories

$$D=\Sigma {\text{pi}}^{2}$$

$$B=1/\Sigma {\text{pi}}^{2}$$

$$H^{\prime }=‐\Sigma p_{i}\log10\;p_{i}$$

Ba=(B‐1)/(S‐1)

$$J^{\prime }=H^{\prime }/\log10\;S$$

where *pi* is the biomass proportion of a given food category in the total biomass of all food categories.

To compare diversity indices in two fish size classes, a bootstrap procedure was used. All these analyses were conducted using the PAST v3.15 software (Hammer et al. 2001).

To estimate diet overlap, the Schoener α index and the Horn Ro index were used. These indices were defined as:

$$\alpha =1‐0.5\;\Sigma |p_{\text{ix}}{\;\text{‐ p}}_{\text{iy}}|$$

$$\text{Ro}=[\Sigma \left(p_{\text{ix}}+p_{\text{iy}}\right)\log\left(p_{\text{ix}}+p_{\text{iy}}\right)‐\Sigma p_{\text{ix}}\log\;p_{\text{ix}}‐\Sigma p_{\text{iy}}\log\;p_{\text{iy}}]/2\log2$$

where p_ix_ and p_iy_ are the proportions of the ith food resource used by the xth and yth class of specimens. The Schoener α is the most commonly used niche overlap measure but the Horn Ro is considered the best measure of dietary overlap (the lowest bias as sample size increases) when food items are expressed as abundance instead of individual numbers (Smith and Zaret 1982). Each index alone may be insufficient to identify a relationship between niche overlap and competition, representing resource partitioning (Krebs 1999); thus, both estimates for dietary overlap were used together. Both estimated indices vary between 0, indicating no overlap, and +1, when diets are identical (Wallace 1981). For all indices, average values and their standard errors were obtained using the jackknife technique (Krebs 1999).

Based on our experience and previous studies, weatherfish food items were grouped into 5 broad categories based on prey habitat; that is, benthic invertebrates (BE), epiphytic prey (EP), epiphytic/benthic prey (EP/BE), water column (planktonic prey ‐ PL), and detritus (DE), as a separate food types (Table 1). All individuals were clustered (Hierarchical Cluster Analysis, Euclidean distance, Ward’s method based on the minimum variance criterion) to separate juvenile and mature fish into clusters that are discrete and homogenous with respect to the environmental groups of each food type. The appropriate number of clusters was distinguished by splitting the dendrogram to maximize the heterogeneity of the resulting clusters.

**TABLE 1:** Diet composition of the juvenile and mature weatherfish expressed as food category percentage of biomass %W (mean, **S**tandard **D**eviation and **C**oefficient of **V**ariation), frequency of occurrence (%FO), and relative importance index (%IRI). The food categories were assigned to a habitat type, that is, BE—benthic, EP – epiphytic, EP/BE—epiphytic/benthic, PL—planktonic and DE—detritus.

food categories	habitat type	juveniles					mature				
		%W			%FO	%IRI	%W			%FO	%IRI
		mean	SD	CV			mean	SD	CV		
Detritus	DE	4.00	5.93	148.22	54.55	2.71	20.77	23.35	112.41	100.00	24.65
Copepoda	PL	29.22	16.61	56.85	87.88	42.45	17.86	11.62	65.10	100.00	22.27
Cladocera—Chydoridae	PL	5.70	6.38	112.00	84.85	9.04	1.09	1.99	181.90	73.08	0.57
Cladocera—others	PL	0.03	0.11	404.25	6.06	0.00	0.44	2.13	487.17	7.69	0.10
Ostracoda	BE	3.29	3.57	108.66	81.82	4.90	3.69	3.48	94.17	92.31	3.40
Oligochaeta	BE	3.18	17.39	546.56	12.12	0.06	6.12	18.11	296.12	15.38	2.48
Trichoptera	EP	0.21	0.64	300.99	15.15	0.06	0.66	2.73	413.33	11.54	0.03
*Asellus aquaticus*	EP/BE	19.33	21.33	110.33	84.85	16.30	16.54	12.68	76.62	92.31	21.65
Ephemeroptera	EP	0.47	1.37	293.36	12.12	0.16	0.23	0.84	357.83	7.69	0.03
Zygoptera	EP	0.10	0.60	574.46	3.03	0.00	‐	‐	‐	‐	‐
Coleoptera (larvae)	EP	6.37	8.57	134.54	72.73	6.82	2.99	3.96	132.48	57.69	2.24
Coleoptera (imagines)	EP	‐	‐	‐	‐	‐	0.12	0.62	509.90	3.85	0.01
Gastropoda	EP	4.52	6.72	148.80	69.70	4.89	2.33	4.06	174.24	53.85	1.55
Hirudinea	EP/BE	0.03	0.15	574.46	3.03	0.00	1.25	6.35	509.90	3.85	0.18
*Podura aquatica*	PL	0.00	0.03	574.46	3.03	0.00	‐	‐	‐	‐	‐
Diptera—not Chironomidae	BE	1.14	6.00	530.00	6.06	0.06	0.23	1.18	509.90	3.85	0.01
Heteroptera	EP	0.00	0.22	400.45	6.06	0.01	0.28	1.05	382.04	7.69	0.01
Hydrachnellae	EP	0.05	0.14	272.16	18.18	0.03	0.04	0.10	248.39	15.38	0.01
others	PL	5.25	17.37	330.60	51.52	1.52	0.56	1.50	270.22	26.92	0.21
Chironomidae—Prodiamesinae	BE	0.09	0.33	352.59	9.09	0.02	‐	‐	‐	‐	‐
Chironomidae—Tanypodinae	EP/BE	1.89	3.08	162.64	51.52	1.62	11.97	9.77	81.63	84.62	11.81
Chironomidae—Orthocladiinae	EP	6.40	7.86	122.90	69.70	6.15	2.58	2.48	96.14	80.77	2.56
Chironomidae—Chironomini	BE	4.87	6.00	124.25	72.73	4.83	9.14	6.16	67.33	88.46	9.33
Chironomidae—Tanytarsini	EP/BE	2.73	4.72	172.70	69.70	2.18	0.82	1.22	148.79	53.85	0.50
Chironomidae—pupa	PL	0.24	0.99	408.68	6.06	0.01	0.29	1.07	369.53	11.54	0.02
Detritus	DE	4.13	5.98	144.96	56.25	2.33	20.77	23.35	112.41	100.00	18.81
Benthic	BE	29.20	25.32	86.74	100.00	23.43	27.42	18.92	68.99	100.00	34.02
Epiphytic	EP	21.69	13.60	62.87	87.50	22.25	10.21	8.23	80.66	88.46	8.15
Epiphytic/Benthic	EP/BE	6.04	7.14	118.21	68.75	4.93	21.32	14.49	67.94	92.31	19.66
Planktonic	PL	38.87	19.32	49.70	90.63	47.04	20.23	14.02	69.32	100.00	19.35
others	‐	0.07	0.28	389.49	12.50	0.00	0.05	0.16	338.48	11.54	0.01

To identify differences in the feeding habits of juvenile and mature weatherfish, Discriminant Function Analysis (hereafter DFA) following canonical variate analysis (CVA) was performed on the suite of food types above. This analysis allowed identification of those categories that most contributed to group separation. The abundances of food types were arcsine transformed to meet DFA assumptions. DFA was subsequently performed on variables that differed significantly among fish groups and differentiation of fish groups was determined with Wilks’ λ, F, and P statistics.

## 3 RESULTS

**FIGURE 3:**
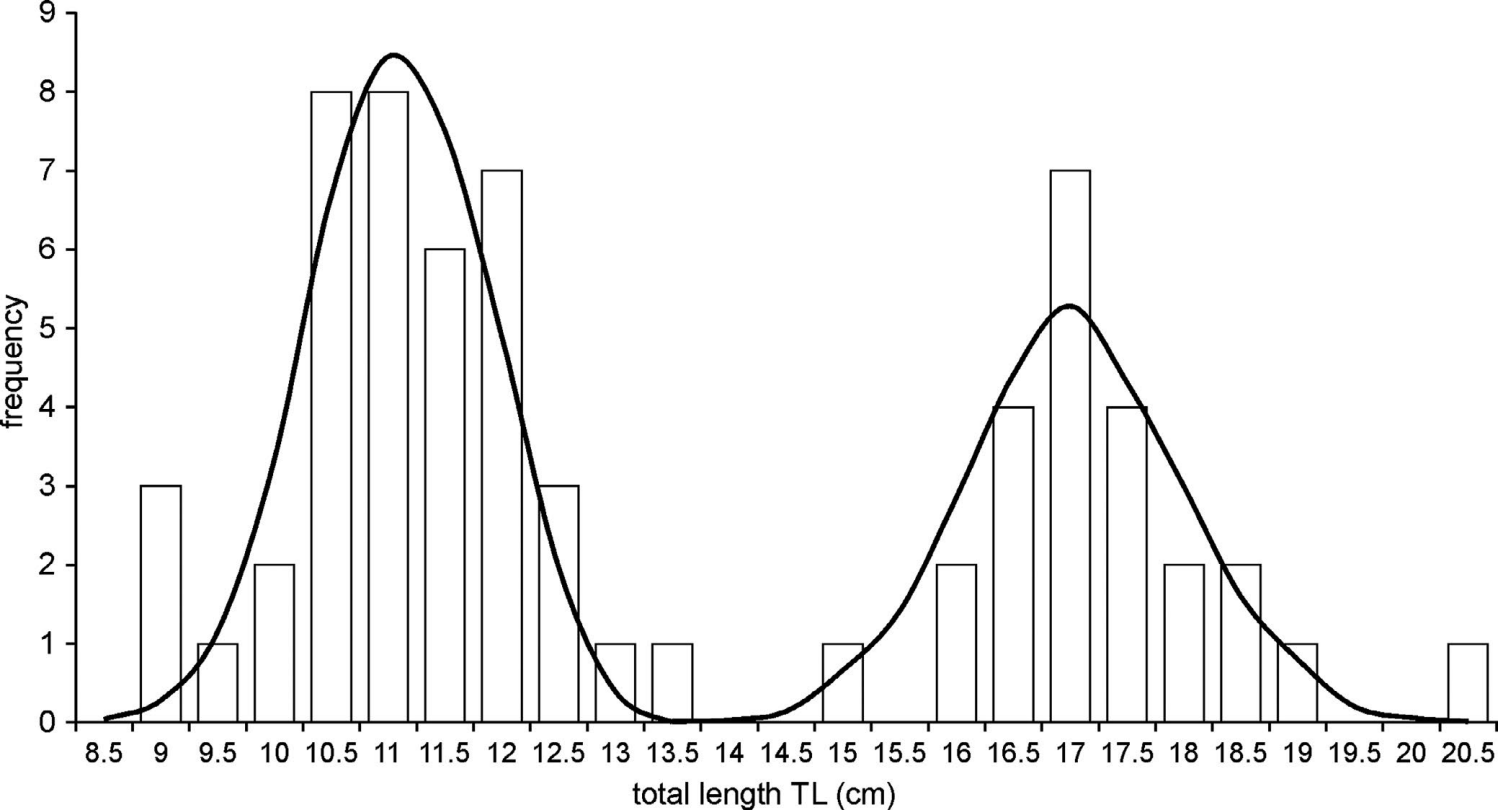
Length‐frequency distribution of weatherfish in the Południowy canal

A total of 64 weatherfish were caught and their length‐frequency distribution showed clear two size classes (Fig. 3), representing small juvenile specimens and larger, mature fish of both sexes. The smaller fish were 11.4 ± 0.71 cm (mean ± sd) TL, whereas mature individuals were 17.3 ± 1.0 cm TL. Although juvenile fish consumed a smaller amount of food (73.98 ± 67.04 mg) than mature fish (142.65 ± 100.62 mg) (t_42_ = 2.99, p > 0.005), both groups did not differ in the fullness coefficient (FC) which related gut content weight to fish weight (1.14 ± 1.11, 0.72 ± 0.70 for juvenile and mature fish, respectively; t_26_ = 1.67, p = 0.101). In the alimentary tracts of dissected fish, 25 major food categories were identified. Weatherfish fed primarily on Copepoda, Cladocera (in particular *Chydorus sphaericus*), Ostracoda, Chironomidae (represented by 4 subfamilies) and Coleoptera larvae, Oligochaeta, Gastropoda, *Asellus aquaticu*s, and detritus (Table 1). The other food categories identified in the diet can be considered as trivial based on their amount and frequency in the diet (Table 1). Variation in the amount of each food category was high in both groups of fish, with the coefficient of variation (CV) exceeding 100% (Table 1).

IRI values also indicated that Copepoda (42.45% IRI) was the most important dietary component for the small size class of weatherfish (Table 1). However, other components of the diet: such as *A. aquaticu*s, Chydoridae, Ostracoda, Coleoptera larvae, Chironomini, Orthocladiinae, Gastropoda, and Tanytarsini, were also consumed with comparable frequency (from 69.70 to 84.85% in diet composition). In the case of mature individuals, the most important dietary component was detritus (24.65% IRI), followed by Copepoda (22.27% IRI), *A. aquaticu*s (21.65% IRI), and Chironomidae larvae: Tanypodinae, Chironomini (11.81% and 9.33% IRI, respectively).

The diet composition and importance of food items differed markedly between size classes (ANOSIM: R statistic = 0.22, p < 0.001). SIMPER analysis showed that dissimilarity in the diet composition of different size classes was due to Copepoda, detritus, *A. aquaticus,* and Tanypodinae (Table 2). These four categories together constituted over 53.64% of cumulative dissimilarity in weatherfish diet between size classes.

**TABLE 2:** Dissimilarity in diet between juvenile and mature weatherfish

food categories		Dissimilarity		Age classes	
	Average	Contribution%	Cumulative %	juvenile	mature
Copepoda	9.63	15.73	15.73	30.10	17.90
Detritus	9.23	15.06	30.79	4.00	20.80
*Asellus aquaticus*	8.52	13.91	44.69	19.00	16.50
Chironomidae—Tanypodinae	5.48	8.95	53.64	1.89	12.00
Oligochaeta	4.44	7.25	60.89	3.18	6.12
Chironomidae—Chironomini	3.72	6.08	66.90	5.03	9.14
Coleoptera (larvae)	3.11	5.08	72.04	6.37	2.99
Chironomidae—Orthocladiinae	2.95	4.82	76.86	6.40	2.58
Cladocera—Chydoridae	2.81	4.59	81.45	5.98	1.09
others	2.68	4.38	85.82	5.22	0.56
Gastropoda	2.50	4.09	89.91	4.46	2.33
Ostracoda	1.89	3.08	92.99	3.23	3.69
Chironomidae—Tanytarsini	1.39	2.27	95.26	2.89	0.82

Both fish size classes consumed a wide spectrum of prey groups but there was no significant difference in food niche width between juveniles and mature fish (Table 3). However, differences in Simpson’s dominance index showed that in mature fish food items were distributed more equally than in juveniles. As a result of similar niche width, diet overlap between size groups was also high (over 0.5) for both indices (Schoener α = 0.54 ± 0.029 and Horn Ro = 0.75 ± 0.063).

**TABLE 3:** Food niche width of juvenile and mature weatherfish. Average values and their standard errors were calculated according to jackknife method. S*—number of food categories expressed as mode and range. Significant difference (permutation p) is indicated in bold

	juveniles		mature		
	average	se	average	se	Perm p
S*	15	2‐23	18	3‐18	*Not tested*
D	0.17	0.04	0.11	0.03	**0.016**
B	4.91	0.36	5.33	0.45	0.407
H'	2.38	0.24	2.52	0.14	0.383
Ba	0.35	0.02	0.36	0.03	0.928
J'	0.64	0.01	0.69	0.03	0.340

**FIGURE 4:**
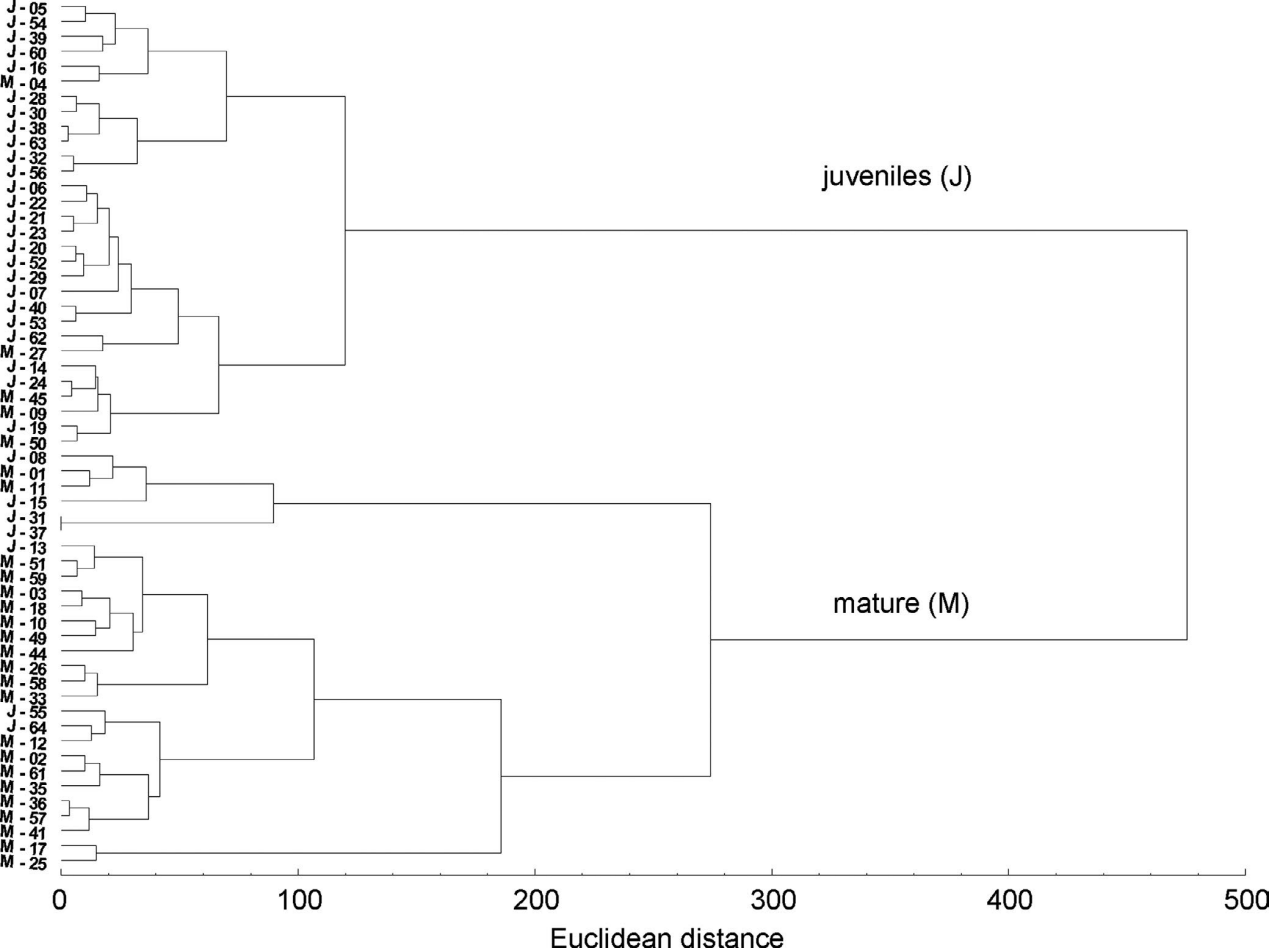
Cluster analysis (Ward method, Euclidean distance) for 64 specimens of juvenile and mature weatherfish based on the amount of food types according to prey habitat use and detritus

Assignment of food items into 5 broad types: that is, benthic, epiphytic, epiphytic/benthic, planktonic, and detritus, revealed differences in diet composition of the fish size classes. For juveniles, the most important dietary component were prey items associated with the water column (38.87%), benthic (29.20%), and epiphytic (21.69 %). In contrast, for mature fish the most important diet components were benthic (27.42%), benthic/epiphytic (21.32%), and detritus (20.77%) (Table 1). Cluster analysis of food types confirmed the distinction of the two groups comprising juvenile and mature individuals (Fig. 4). Only a few individuals were classified incorrectly; that is, 7 juveniles were included in the cluster for mature fish, and 5 mature fish clustering with juveniles (Fig. 4).

Further differences in feeding habits were confirmed by discriminate analysis (Wilks’λ = 0.46, F_5.52_ = 12.10, p<0.001) and to explain these, only one discriminate function was necessary. An overall classification was correct in 87.93% of specimens. Correct classification of both fish groups was in a similar proportion; that is, juvenile fish were correctly classified in 87.5% of cases and mature fish in 88.5%. Wilks’ λ revealed that among the 5 food types, only detritus and epiphytic/benthic prey were included in a discriminant model with a similar correlation with the discriminant axis (Table 4). For mature fish, detritus was one of the most frequently consumed food types and the amount of this food type (as average %) was 5 times more abundant than for juveniles (4.13 ± 5.98 and 20.77 ± 23.35, juvenile and mature specimens, respectively). Similar differences were also noted for epiphytic/benthic prey (6.04 ± 7.14 and 21.32 ± 14.49, juvenile and mature specimens, respectively). Because smaller and larger weatherfish showed a difference in the frequency of occurrence of these two types of food, the IRI for detritus was 8 times more important for mature fish (%IRI = 18.81) than for juveniles (%IRI = 2.33), while the importance of epiphytic/benthic prey was %IRI = 19.66 for mature and %IRI = 4.93 for juvenile weatherfish.

**TABLE 4:** Correlations of food types with canonical axes 1 from discriminant function analysis and their contribution to discrimination among juvenile and mature weatherfish. Amount food types expressed as percentage was arcsine transformed. Significant differences are indicated in bold

food type	Axis 1	Wilks' λ	F _1. 52_	p
Detritus	‐0.64	0.51	**5.45**	**0.023**
Benthic	0.05	0.47	0.48	0.490
Epiphytic	0.39	0.46	0.12	0.733
Epiphytic/Benthic	‐0.65	0.62	**18.36**	**0.000**
Planktonic	0.41	0.462	0.00	0.943
Eigenvalue	1.16			

## 4 DISCUSSION

The aim of the present study was to define weatherfish diet composition, feeding habits, and possible ontogenetic niche shift in a typical habitat for the species from a region where the fish is still relatively abundant. Weatherfish proved to be opportunistic feeders, using the most readily available food resources. At the study site, weatherfish fed on a wide spectrum of food categories, though the diet was dominated by zooplankton (Copepoda), *A. aquaticus*, the larvae of macroinvertebrates (Chironomidae), and detritus. Food items of animal origin constituted almost 90% of the total weight of the gut contents. Our results demonstrate that under favorable conditions diet composition was much more broader than noted in the previous studies (Boroń et al. 2002; Pyrzanowski et al. 2019), although the main prey groups; that is, invertebrate larvae, zooplankton, and detritus, matched previous observations (Boroń et al. 2002; Pyrzanowski et al. 2019). However, none of these studies reported the possible ontogenetic shift in diet. The few studies on ontogenetic shifts in the feeding patterns of *Misgurnus* species were conducted for the oriental weatherfish (*Misgurnus anguillicaudatus*), which is closely related to *M. fossilis*. Like weatherfish, *M. anguillicaudatus* was originally defined as a typical detritus feeder (Watanbe and Hidaka 1983), but later study demonstrated that it feeds mainly on small benthic invertebrates (Tabor et al. 2001; Kanto et al. 2003; Urquhart and Koetsier 2014). In its native range, smaller individuals feed mainly on zooplankton (Kubota 1961) and small benthic invertebrates, such as Ephermeroptera, Trichoptera and Chironomidae larvae (Katano et al. 2003). Larger individuals (at approximately 10‐13 cm TL) tend to undergo an ontogenetic diet shift, switching to a herbivorous diet based on organic debris (Kubota 1961).

Changes in habitat preferences and switching to a different food type during ontogenesis is common in many fish species. Changes in the diet are associated with changes in body size and habitat occupancy, probably arising from an inability to optimally use the resources of previously occupied habitats. Ontogenetic shifts in prey preference are also associated with increased energetic requirements associated with a larger body size. Furthermore, large individuals often show a more diversified diet, indicating a capacity to exploit a broader range of prey (Werner and Gilliam 1984; Labropoulou et al. 1997). In many fish species, an increase in size is associated with a concomitant change in diet preference, with a commonly observed transition from small food items, such as phytoplankton or zooplankton, to much larger prey, such as macroinvertebrates (Nunn et al. 2007). A dietary shift could also be explained as behavioral response to maturation (Labropoulou et al. 1997). Our results demonstrated that the diet composition of the two size classes reflected possible ontogenetic changes in prey preference. All analyses indicated differences between small (TL≤12 cm) and large (TL>12 cm) weatherfish, which may coincide with sexual maturity (Pyrzanowski et al. 2020b).

Classification of prey to their appropriate ecological groups (benthos, epiphyton and zooplankton) indicated that smaller weatherfish (identified as juveniles) foraged mostly from the water column and plants, while larger individuals (age 3+ and older) fed with similar frequency from the substrate. The presence of zooplankton, in particular Copepoda, was particularly conspicuous in the small size class and constituted the most numerous and most important component of the diet of juveniles. Although copepods move rapidly and are relatively difficult to catch they are a natural prey of virtually all fish larvae (McKinnon et al. 2003). Another representative of the zooplankton, which has been found to contribute significantly to the diets of small fish, was the common *C. sphaericus* (Chydoridae, Cladocera), which is relatively small and tolerant of extreme environmental conditions (Belyaeva and Deneke, 2007). *C. sphaericus* was also an important part of the diet of large weatherfish, but was less frequent. Due to their limited motor skills, Cladocera prefer lentic habitats, typically inhabiting submerged plants and macroalgae of the littoral zone (Adamczuk 2014). The presence of Copepoda and Chydoridae in the diet of small weatherfish indicates that they feed mainly among vegetation and in the water column. Smaller individuals also tend to consume infrequently encountered food items from the water column that were of terrestrial origin.

An important component of the diet for both weatherfish size groups was *A. aquaticus,* the most common freshwater Isopoda in European waterbodies. Due to its eurybiotic lifestyle, it occurs in a large variety of habitats (Sworobowicz et al. 2015). The species is highly tolerant of organic pollution and has been used as an indicator of water quality (Whitehurst 1991). It is a species associated with the substrate, particularly with decomposing plant material on which it feeds (Sworobowicz et al. 2015). At the study site, *A. aquaticus* probably occurs both on the canal substrate, but also on plants, which in summer at the peak of growing season may fill the entire watercourse. Given its likely ubiquity, it is unsurprising that *A. aquaticus* was consumed to a comparable degree by both small and large weatherfish, foraging in different zones of the study site.

Among the chironomids, two general forms of larvae: pelophilous and phytophilous are distinguished. The first group includes taxa inhabiting bottom sediments, while the second is associated with macrophytes (Armitage et al. 1995). Our research shows that large fish fed mainly on relatively large benthic forms: *Chironomus*, which are opportunistic tube dwelling deposit feeders (De Haas et al. 2006), and predatory Tanypodinae (*Psectrotanypus*, *Procladius*), which are usually free swimming (Vallenduuk and Pilot 2013). In contrast, smaller weatherfish consumed mainly *Cricotopus*, *Corynoneura* (Orthocladiinae), and *Paratanytarsus* (Tanytarsini), typically inhabiting macrophytes (Verdonshot and Lengkeek 2009; Čerba et al. 2010).

Detritus and organic debris can be an important source of nutrients and organic dietary components, such as carbon and nitrogen (Urquhart and Koetsier 2014), but as a primary food source is typically lower in energy and protein relative to invertebrate prey (Bowen et al. 1995). In this study, mature specimens were much more likely to consume detritus than juveniles, with detritus comprising up to about 20% of the diet of mature weatherfish. Assuming that the substrate is the preferred habitat of mature weatherfish (Meyer and Hinrichs 2000; Boroń et al. 2002; Kottelat and Freyhof 2007; Pekarik et al. 2008), detritus may occur in the diet of larger individuals as a core component of the diet, and also as an unintentional side effect of within‐substrate feeding on invertebrate prey (Urquhart and Koetsier 2014). For smaller weatherfish, which tend to occupy submerged plants, possibly as a result of competition from larger individuals, access to detritus as a food source may be more limited. Alternatively, smaller individuals may actively seek more energetically valuable animal components in their diet. Notably, in unfavorable environmental conditions, at elevated temperatures and low dissolved oxygen concentrations, detritus comprises the primary food resources for the full size spectrum of weatherfish (Pyrzanowski et al. 2019). A similar relationship, indicating an ontogenetic shift in diet, can be seen in the case of *M. anguillicaudatus* in which large fish tended to be detritivorous (Kubota 1961).

Though we recorded differences in the feeding pattern of juvenile and mature weatherfish, considerable diet overlap was noted. Both Schoener’s and Horn’s index excide the value 0.6, which is usually considered significant (Wallace 1981). Differences between these indices could result from unjustified assumptions. Given that the proportion of food items was calculated based on weight, Horn’s measurement is more appropriate than Schoener’s (Krebs 1999). Occasionally R values derived from ANOSIM are used to assess diet overlap in animal food resource utilization (Creque and Czesny 2012). In these cases, R values of 0.25 are considered to represent substantial overlap, values 0.26–0.5 moderate overlap, and values >0.5 little to no overlap in prey use (Creque and Czesny 2012). In our study R = 0.22, potentially indicating important food niche overlap between fish size groups. However, the overlap measures do not necessarily indicate competition between juveniles and mature fish, especially when food resources are abundant. Differences in dietary composition between juveniles and mature weatherfish revealed by DFA, as well as cluster analysis (Fig. 4), result from microhabitat use than food resources partitioning.

In conclusion, a detailed analysis of the diet of weatherfish in a canal system indicated that this species in an opportunistic feeder and showed a change in feeding site affinity with size, from a diet derived from the water column in juveniles to one associated with the substrate in mature adults. It was also shown that the resolution of prey identification, and assigning prey to specific habitats, is critical for understanding the allocation of food resources. A switch between a benthic and pelagic (zooplanktonic) diet is usually related to the feeding efficiency for particular prey types and occurs during ontogeny (Lammens and Hoogenboezem 1991). In the case of weatherfish, differences in feeding mode; that is, benthic foraging by mature specimens and pelagic/epiphytic by juveniles may arise from intraspecific competition for resources.

## CONFLICT OF INTEREST

The authors declare that they have no known competing financial interests or personal relationships that could have appeared to influence the work reported in this paper.

## AUTHOR CONTRIBUTION

**Kacper Pyrzanowski:** Conceptualization (lead); Formal analysis (equal); Investigation (equal); Methodology (equal); Visualization (lead); Writing‐original draft (lead). **Grzegorz Zięba:** Conceptualization (supporting); Investigation (equal). **Joanna Leszczyńska:** Investigation (equal); Visualization (supporting). **Małgorzat Adamczuk:** Investigation (equal). **Małgorzata Dukowska:** Investigation (equal); Methodology (supporting). **Mirosław Przybylski:** Conceptualization (supporting); Formal analysis (equal); Investigation (equal); Methodology (equal); Supervision (lead).

## ETHICAL APPROVAL

The weatherfish is protected in Poland, therefore, all procedures were carried out under permission from the Local Ethics Committee (66/ŁB729/2014) and the Regional Directorate of Environmental Protection (WPN‐II.6401.268.2014.KW2).

## Data Availability

All data from manuscript are accessible in the Dryad digital repository (https://doi.org/10.5061/dryad.bvq83bk80)
